# Investigating the presentation of uncertainty in an icon array: A randomized trial

**DOI:** 10.1016/j.pecinn.2021.100003

**Published:** 2022-12

**Authors:** Gabriel Recchia, Alice C.E. Lawrence, Alexandra L.J. Freeman

**Affiliations:** Winton Centre for Risk and Evidence Communication, Department of Pure Mathematics and Mathematical Statistics, University of Cambridge, Cambridge, UK

**Keywords:** Risk communication, Icon arrays, Pictographs, Pictograms, Uncertainty, Communication, Genetic risk communication

## Abstract

**Background:**

Clinicians are often advised to use pictographs to communicate risk, but whether they offer benefits when communicating risk imprecision (e.g., 65%-79%) is unknown.

**Purpose:**

To test whether any of three approaches to visualizing imprecision would more effectively communicate breast and ovarian cancer risk for *BRCA1* pathogenic variant carriers.

**Methods:**

1,300 UK residents were presented with a genetic report with information about *BRCA1*-related risks, with random assignment to one of four formats: no visualization (text alone), or a pictograph using shaded icons, a gradient, or arrows marking range endpoints. We also tested pictographs in two layouts. Analysis of variance (ANOVA) and regression was employed.

**Results:**

There was no effect of format. Participants shown pictographs vs. text alone had better uptake of breast cancer risk messages (*p* < .05, *η*^*2*^ = 0.003). Pictographs facilitated memory for the specific amount of risk (*p* < 0.001, *η*^*2*^ = 0.019), as did the tabular layout. Individuals not having completed upper secondary education may benefit most.

**Conclusions:**

We found weak evidence in favor of using simple pictographs with ranges to communicate *BRCA* risk (versus text alone), and of the tabular layout.

**Innovation:**

Testing different ways of communicating imprecision within pictographs is a novel and promising line of research.

## Highlights

• Clinicians are often encouraged to convey uncertainties and to use visualizations.
• We did not find a clear benefit of any specific method of visualizing a risk range.
• Icon arrays with ranges may improve gist and verbatim knowledge vs. text alone.
• Icon arrays in tabular layouts may also improve verbatim knowledge.

## 1 Introduction

Research suggests that many clinicians have not been provided sufficient tools, training or support to communicate accurately about genetic risk [1,2], and that there is substantial room for improvement in how information is communicated within genetic reports [[2], [3], [4]]. This has led to efforts to improve the effectiveness of the communication by making genetic reports easier to understand for clinicians and patients [2,[4], [5], [6], [7], [8], [9], [10], [11], [12], [13]]. Although primary care clinicians are accustomed to communicating about risks and uncertainties in general terms, genetic reports pose unique challenges. For example, statistics in genetic reports are sometimes even difficult for clinicians to comprehend [14,15], let alone communicate to a patient.

The most important number to appear on a genetic report may be the percentage of individuals with the result who can be expected to experience a particular health outcome, which is often uncertain. In the East of England, genetic counsellors describe the lifetime breast cancer risk to carriers of *BRCA* pathogenic variants with deliberate imprecision (e.g., 65%-79%) in order to communicate that there is uncertainty around the precise level of risk[16]. Healthcare professionals have long been encouraged to use icon arrays, also known as pictographs, to help communicate percentages[17,18]. However, the best approach for communicating imprecision within an icon array is unclear. Icon arrays have been developed using techniques such as partially filled icons[19] and icons that fade from one color to another[20], but comprehension of such representations has not been broadly empirically tested. The present study investigated comprehension of three visualizations for communicating imprecision in the context of genetic risk, as well as of text alone.

### 1.1 Background

Compared to numerical information presented as text alone, icon arrays often facilitate more accurate recall and comprehension of risks[[21], [22], [23], [24], [25], [26], [27]]. Cognitive psychologists distinguish between specific ‘verbatim’ information (e.g., “the risk of experiencing X is 62%”) and the essential point or ‘gist’ (e.g., “the risk is higher than average”), as storage and retrieval of verbatim and gist information occur via different cognitive pathways[28]. In laboratory and clinical settings, icon arrays have been shown to communicate both forms of information adequately, with some evidence that they may be particularly helpful for low-numeracy participants[22,23,29]. That said, one clinical study found poorer short-term verbatim recall when frequencies were supplemented with icon arrays as compared to percentages or frequencies alone[30], but there were no significant differences with respect to gist knowledge. A systematic review of methods for communicating probabilistic evidence to patients concluded that "icon arrays and bar graphs both lead to improvements in accuracy and comprehension, with neither being clearly superior"[27]. Investigations of feelings towards icon arrays have yielded mixed results regarding preferences and trustworthiness[18,23,27,31], although several studies found that they are viewed as “helpful” or “useful”[18,32,33]. This is affected by details such as the type of icon used in the visualization[34,35], which may account for some of the variation in findings.

Risk visualizations have also been investigated in the context of communicating *BRCA*-associated risk. Some studies have found that visualizations are valued by carriers of *BRCA1/2* pathogenic variants[36,37] and result in higher decision satisfaction[38], but have found no clear differences in comprehension, well-being or treatment intentions[38,39]. Studies communicating breast cancer risk with ‘incremental’ icon arrays containing three or more colors found that these have not fared as well as other formats[36,37,40], in line with research finding that this kind of icon array can be difficult to interpret without training[41]. *BRCA* risk communication studies using simpler icon arrays have been inconclusive[39,42] or have reported benefits[43,44].

There has been work related to communicating imprecision [31,[45], [46], [47], [48], [49]], but research explicitly testing approaches to communicating imprecision using icon arrays is limited. Two exceptions are [50], which found that more people understood imprecision when it was described qualitatively than when it was visualized using an icon array with a gradient overlay, and [51], which found no differences in recall of information whether imprecision was communicated within a table, icon array, or bar graph.

### 1.2 Aims of the communication

Simple icon arrays may help patients come away with a better understanding of risks to their health, but it is unclear whether this remains true if they are made more complicated with the inclusion of a risk ‘range’. Our primary aim was to determine which of the approaches in Table 1 best communicated the basic messages that breast and ovarian cancer risk are higher for medically unmanaged *BRCA1* pathogenic variant carriers than for the general population, and that breast cancer is more probable than ovarian cancer. Additional questions of interest included whether any of these approaches affected participants’ understanding of the specific amount of risk that carriers face, how they felt about the risk, and their perceptions of how easy the risks were to remember and understand.

**Table 1:** Stimuli displayed in the four format conditions that participants were randomized to. Each of the examples here illustrate the statement “About 2 to 5 out of 8 women with alterations like yours in *BRCA1* (with no treatment) develop ovarian cancer”.

Condition	Description
Arrows (endpoint marking)	• Those expected to experience the outcome are in black and lie to the left of the arrows • Those expected not to experience it are in light gray and lie to the right • Those within the uncertainty range are in black and lie underneath the arrows
Gradient	• The proportion expected to experience the outcome appear in black • Those expected not to experience it appear in light gray • Those within the uncertainty range are illustrated with a gradient fading from black to light gray
Shaded	• The proportion expected to experience the outcome appear in black • Those expected not to experience it appear in light gray • Those within the uncertainty range appear in an intermediate shade of gray
No visualization	• Control condition: no visualization displayed • Risks were still communicated within the text on the genetic report template (as in the above three conditions)

## 2 Methods

### 2.1 Participants

In previous research using a similar gist knowledge measure[52], we found that changes to a bar chart improved gist knowledge with an effect size of d = 0.23. A power analysis with this effect size suggested that 1300 participants would be required to achieve 95% power on the primary analysis, and 80% power on post-hoc equivalence tests. 1300 participants were therefore recruited by the ISO-accredited polling company Respondi via an online survey panel during April 20-25, 2020, quota-sampled so as to be proportional to the UK with respect to age, gender, and education. UK residents aged 18+ were eligible. The study time was estimated at 15 minutes and participants were given the option to be paid £2.18 or to have £2.18 donated to a cancer charity. All were provided with a participant information sheet, completed online written consent forms, and completed the study online. The study was overseen by the Psychological Research Ethics Committee at the University of Cambridge (PRE.2018.077, amended 8 April 2020).

### 2.2 Stimuli

#### 2.2.1 Development of stimuli

Rather than relying only upon our own intuitions to select which visualizations to evaluate, we solicited feedback from participants in a user-centered design exercise that we conducted to develop a set of patient-centered genetic reports. This involved one-on-one interviews with 13 healthcare professionals, 16 individuals who had undergone *BRCA* testing, and 13 laypersons with other backgrounds, ranging from people with family members who had undergone *BRCA* testing to people with no knowledge of genetic testing at all. Each participated in one of four rounds of interviews, and visualizations were modified in response to feedback after each round. The protocol consisted of questions about a range of topics, with the final prompts relating to the visualizations. This provided insights into preferences and possible misinterpretations. Ultimately, we selected three particularly promising visualizations to test quantitatively.

Because this work was conducted in the context of a multi-stakeholder project to develop reports intended for clinical use, our choices were subject to certain constraints. For example, to avoid confusion with existing practices describing population lifetime breast cancer risk as “1 out of 8”, arrays were constructed with eight icons. This meant that population lifetime ovarian cancer risk had to be represented with a fraction of an icon. Although these constraints impose limitations on the interpretation of our findings, we decided that adhering to them would increase the probability that any results would hold in our real-world use case, and aid quick implementation into clinical use.

#### 2.2.2 Description of stimuli

**Fig. 1:**
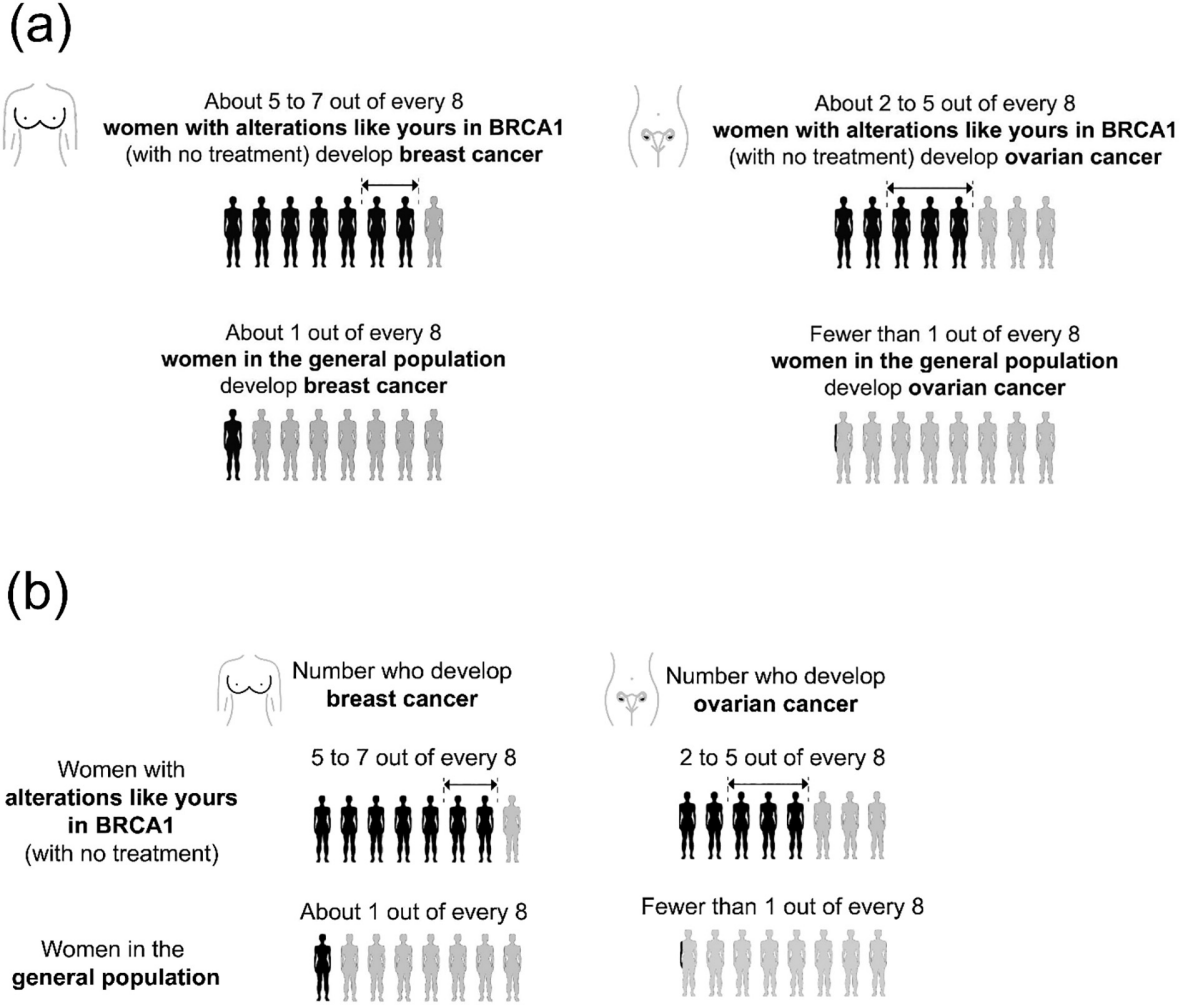
(a) The self-contained layout. (b) The tabular layout.

Each participant was shown a genetic report (Figure S1). For some participants, the report included an icon array (see Table 1 for descriptions and images). The genetic report communicated information about the recipient’s risk of breast and ovarian cancer. The recipient was a fictional woman who had received a positive *BRCA1* test result. Irrespective of format, each icon array also appeared in either a ‘self-contained’ or ‘tabular’ layout, which differed in the placement and content of labels, as illustrated in Fig. 1.

### 2.3 Measures

The primary dependent variables were three measures of gist knowledge. These investigated whether participants correctly understood that, for a woman with a pathogenic *BRCA1* alteration, (1) breast cancer is more likely than ovarian cancer; (2) breast cancer risk is increased; and (3) ovarian cancer risk is increased. These were adapted from a measure of gist knowledge validated in previous research[52]. Analyses of these measures were preregistered at https://osf.io/znjh9. We also collected exploratory dependent variables: verbatim knowledge, attitudes towards the risk information, subjective understanding of the risk information, subjective risk assessment, and subjective recall (see Table 2 for detailed description of measures), as well as covariates: objective health literacy, subjective health literacy, numeracy, and personal experience with cancer. Objective health literacy was measured using the UK version of Newest Vital Sign[53], subjective health literacy with subscale 9 of the Health Literacy Questionnaire[54], and numeracy with the adaptive Berlin Numeracy Test[55].

**Table 2:** Summary of dependent measures on survey. For multiple-choice questions, any answer with an asterisk was counted as correct. All verbatim knowledge questions contained the additional instruction “If you can't remember, just make your best guess.”

Construct	Measure
Gist knowledge measure 1: breast cancer more likely than ovarian	Maximum of 4 points, calculated as follows: 1 point for correct answer to “Is a woman with a gene alteration like Carla's more likely to get **breast cancer** or **ovarian cancer** during her lifetime?” [Much more likely to get breast cancer*; More likely to get breast cancer*; Somewhat more likely to get breast cancer*; Exactly the same chance of getting breast cancer or ovarian cancer; Somewhat more likely to get ovarian cancer; More likely to get ovarian cancer; Much more likely to get ovarian cancer; I don't know]. 1 point for correct answer to “Which of these two cancers is someone with a gene alteration like Carla's more likely to get: **breast cancer** or **ovarian cancer**?” [Breast cancer*; ovarian cancer; I don’t know] 1 point if answer to “**Out of every 8 women** with **gene alterations like Carla's**, roughly how many will develop **breast cancer** during their lifetimes (if they do not have treatment to reduce their risk)?” is higher than answer to “**Out of every 8 women** with **gene alterations like Carla's**, roughly how many will develop **ovarian cancer** during their lifetimes (if they do not have treatment to reduce their risk)?” Answers were entered in text boxes. 1 point if answer to “About what percentage of women with gene alterations like Carla's develop **breast cancer** (if they do not have treatment to reduce their risk)?” is higher than answer to “About what percentage of women with gene alterations like Carla's develop **ovarian cancer** (if they do not have treatment to reduce their risk)?” Answers were entered using sliders ranging from 0-100. ω_total_ = 0.70
Gist knowledge measure 2: breast cancer risk is increased	Maximum of 4 points, calculated as follows: 1 point for correct answer to “Is a woman's chance of getting **breast cancer** during her lifetime higher if she has a gene alteration like Carla's, or if she is a member of the general population?” [Much higher if she has a gene alteration like Carla's*; Higher if she has a gene alteration like Carla's*; Somewhat higher if she has a gene alteration like Carla's*; Exactly the same chance if she has a gene alteration like Carla's or if she is a member of the general population; Somewhat higher if she is a member of the general population; Higher if she is a member of the general population; Much higher if she is a member of the general population; I don't know] 1 point for correct answer to “Which of these two groups of people is more likely to get **breast cancer**: **people with a gene alteration like Carla's** or **people in the general population**?” [People with a gene alteration like Carla's*, People in the general population, I don’t know] 1 point if answer to “**Out of every 8 women** with **gene alterations like Carla's**, roughly how many will develop **breast cancer** during their lifetimes (if they do not have treatment to reduce their risk)?” is higher than answer to “**Out of** every **8 women** in the **general population**, roughly how many will develop **breast cancer** during their lifetimes?” Answers were entered in text boxes. 1 point if answer to “About what percentage of women with gene alterations like Carla's develop **breast cancer** (if they do not have treatment to reduce their risk)?” is higher than answer to “About what percentage of women in the general population develop **breast cancer**?” Answers were entered using sliders ranging from 0-100. ω_total_ = 0.70
Gist knowledge measure 3: ovarian cancer risk is increased	As in above (Gist knowledge measure 2), but with “ovarian” in place of “breast” throughout ω_total_ = 0.61
Proposed frequency subscale of verbatim knowledge measure	Scale computed on the basis of participants’ answers to four questions; textboxes were provided for participant responses. Maximum of 4 points, calculated as follows: 1 point for correct answer to: “**Out of every 8 women** with **gene alterations like Carla's**, roughly how many will develop **breast cancer** during their lifetimes (if they do not have treatment to reduce their risk)?” If participant provided a single number, it was counted as correct if it fell within the range communicated on the report (inclusive). If participant provided a range, it was counted as correct if its midpoint fell within the range communicated on the report. 1 point for correct answer to: “**Out of every 8 women** with **gene alterations like Carla's**, roughly how many will develop **ovarian cancer** during their lifetimes (if they do not have treatment to reduce their risk)?” If participant provided a single number, it was counted as correct if it fell within the range communicated on the report (inclusive). If participant provided a range, it was counted as correct if its midpoint fell within the range communicated on the report. 1 point for correct answer to: “**Out of** every **8 women** in the **general population**, roughly how many will develop **breast cancer** during their lifetimes?” 1 point for correct answer to: “**Out of** every **8 women** in the **general population**, roughly how many will develop **ovarian cancer** during their lifetimes?” ω_total_ = 0.45
Proposed percentage subscale of verbatim knowledge measure	Scale computed on the basis of participants’ answers to four questions; sliders ranging from 0%-100% were provided for participant responses. Maximum of 4 points, calculated as follows: 1 point for correct answer to: “About what percentage of women with gene alterations like Carla's develop **breast cancer** (if they do not have treatment to reduce their risk)?” Counted as correct if it fell within the range communicated on the report (inclusive). 1 point for correct answer to: “About what percentage of women with gene alterations like Carla's develop **ovarian cancer** (if they do not have treatment to reduce their risk)?” Counted as correct if it fell within the range communicated on the report (inclusive). 1 point for correct answer to: “About what percentage of women in the general population develop **breast cancer**?” Answers between 10% and 15% were counted as correct. 1 point for correct answer to: “About what percentage of women in the general population develop **ovarian cancer**?” Because the report stated only that “fewer than 1 out of every 8 will develop ovarian cancer”, answers lower than 12.5% but not equal to zero were counted as correct. ω_total_ = 0.46
Verbatim knowledge measure	Sum of “Proposed frequency subscale of verbatim knowledge measure” and “Proposed percentage subscale of verbatim knowledge measure”. Maximum of 8 points. ω_total_ = 0.63
Subjective recall	“How easy or difficult was it to remember the information about the risks of breast and ovarian cancer?” [Very difficult, difficult, somewhat difficult, neither easy nor difficult, somewhat easy, easy, very easy]
Subjective understanding	“How easy or difficult is it to understand the information in this section?”, with image of “What This Result Means For You” section (Figure S1) visible [Very difficult, difficult, somewhat difficult, neither easy nor difficult, somewhat easy, easy, very easy]
Subjective risk assessment	“If Carla does not receive treatment to reduce her chances of developing cancer, how likely is it that... **Carla** will develop **breast cancer** sometime during her lifetime?” [Very unlikely, unlikely, somewhat unlikely, neither likely nor unlikely, somewhat likely, likely, very likely]; “**Carla** will develop **ovarian cancer** sometime during her lifetime?” [as above]
Attitudes towards risk information (modified from Marteau, Dormandy & Michie[70])	"If you were in Carla's situation, would you say that seeing the information in this section is:", average of seven 7-point semantic differentials (final two items reverse coded), with image of “What This Result Means For You” section (Figure S1) visible: harmful-beneficial, unimportant-important, bad thing-good thing; unpleasant-pleasant; useless-useful; clear-unclear; helpful-unhelpful.

When treated as unidimensional scales, ω_total_ [56], a measure of reliability whose values have a similar interpretation to Cronbach’s alpha but which is robust to non-normal data, was computed for the measures of gist and verbatim knowledge (values reported in Table 2). For verbatim knowledge, ω_total_ was beneath conventional standards of acceptability for the two proposed 4-item percentage and frequency subscales^1^. Analyses of the proposed subscales are reported for completeness, but readers may wish to take reliability into consideration when deciding how much weight to place on these results.

### 2.4 Design and procedure

**Fig. 2:**
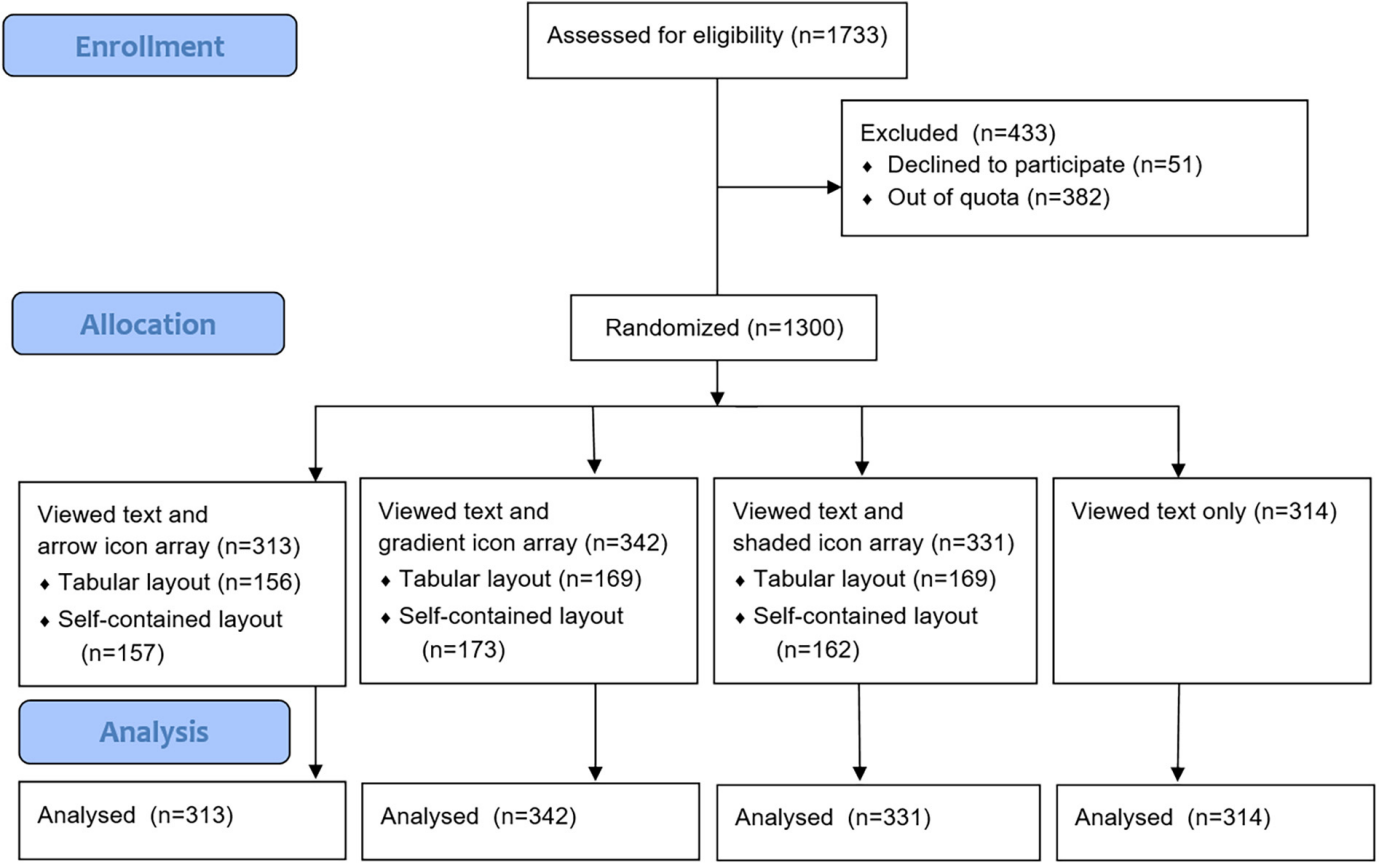
CONSORT flow diagram.

The study used a between-subjects design in which participants were randomized to view a genetic report corresponding to one of the four *format* options in Table 1. Participants shown an icon array were also randomized to view it in the self-contained or tabular layout. A participant flow diagram is given in Fig. 2.

Participants were provided with background information about predictive genetic tests for cancer risk genes from an NHS website and presented with a fictional scenario in which a woman’s mother was diagnosed with a *BRCA1*-related cancer, and the woman (“Carla”) decided to receive a *BRCA1* test. The scenario did not specify whether the mother’s cancer was breast or ovarian. Participants were then randomized by the survey software into one of the four format conditions (and, for those not in the “no icon array” format, into one of the two layout conditions) and viewed the corresponding report. They then completed a questionnaire containing the measures in the following order: attitudes towards risk information, subjective understanding, subjective risk assessment, gist knowledge, verbatim knowledge, subjective recall, health literacy, numeracy, and demographic information.

### 2.5 Data analysis

The primary method of analysis employed was ANOVA, with nonparametric alternatives used in cases where residuals exhibited serious violations of normality (Kruskal-Wallis tests for one-way analyses; aligned ranks ANOVA[57] for two-way analyses). ANOVAs evaluating the effect of format on gist knowledge measures were preregistered. We also preregistered our intent to test whether lay participants’ recall of the increased risk that accompanies a pathogenic *BRCA* variant is improved by the inclusion of an icon array using *any* of the three approaches to illustrating a risk range, although we did not preregister an analysis for doing so beyond that described above. We therefore also tested whether the presence vs. absence of an icon array facilitated gist knowledge, using the same methodology as the primary analysis but collapsing across icon array conditions (i.e., *shaded*, *gradient*, and *arrow* collapsed into a single ‘icon array present’ condition). We explored the impact of individual factors on gist knowledge with exploratory regressions and aligned ranks ANOVAs. Exploratory ANOVAs (or nonparametric alternatives as appropriate) were also used to look for effects of icon array formats on verbatim knowledge, subjective recall, subjective understanding, subjective risk assessment, and attitudes towards risk information.

## 3 Results

Participant demographics, numeracy, and health literacy are summarized in Table 3. Most reported no personal experience with cancer. Participants varied widely on health literacy. Numeracy was negatively skewed, as is typical in general population samples[55]. Kruskal–Wallis tests were conducted to determine whether gist knowledge varied by icon array format (arrows, shaded, gradient, or none); there was no significant difference (Table 4, Table 5).

**Table 3:** Participant demographics, numeracy, and health literacy.^a^, ^b^, ^c^

	N	%	% in UK adult population
Gender			
Female	665	(51%)	51% a
Male	635	(49%)	49% a
Age group			
18-24 years	137	(11%)	12% b
25-34 years	197	(15%)	17% b
35-44 years	197	(15%)	18% b
45-54 years	222	(17%)	17% b
55-64 years	214	(16%)	15% b
65-74 years	237	(18%)	11% b
75+ years	95	(7%)	10% b
Missing	1	(0.1%)	
Education			
Below upper secondary	287	(22%)	19% c
Upper secondary	548	(42%)	46% c
Above upper secondary	465	(36%)	36% c
Do you have any personal experience with cancer?			
Yes	366	(28%)	
No	901	(69%)	
Prefer not to say	33	(3%)	
Numeracy (Berlin Numeracy Score)			
1	662	(51%)	
2	376	(29%)	
3	120	(9%)	
4	142	(11%)	
Objective health literacy (Newest Vital Sign)			
0	139	(11%)	
1	113	(9%)	
2	140	(11%)	
3	156	(12%)	
4	228	(18%)	
5	296	(23%)	
6	228	(18%)	
Subjective health literacy (Health Literacy Questionnaire subscale 9)			
1 (< 1.5)	7	(1%)	
2 (1.5 – 2.49)	42	(3%)	
3 (2.5 – 3.49)	281	(22%)	
4 (3.5 – 4.49)	697	(54%)	
5 (4.5 – 5.0)	271	(21%)	
Missing	2	(0.2%)	

a *Male and female populations: GOV.UK ethnicity facts and figures*, 2018. Available at: https://www.ethnicity-facts-figures.service.gov.uk/uk-population-by-ethnicity/demographics/male-and-female-populations/latest. Accessed June 18, 2019.

b Percentages calculated from *Age groups: GOV.UK ethnicity facts and figures*, 2019. https://www.ethnicity-facts-figures.service.gov.uk/uk-population-by-ethnicity/demographics/age-groups/latest. Accessed June 18, 2019.

c OECD. Table 21.1 - Educational attainment of 25-64 year-olds (2017): Percentage of adults with a given level of education as the highest level attained", in *The Output of Educational Institutions and the Impact of Learning*, OECD Publishing, Paris, 2018: 54. https://doi.org/10.1787/eag-2018-table14-en. Following up on methodology referenced in table notes, and combining this with UK-specific definitions of ISCED levels (http://gpseducation.oecd.org/Content/MapOfEducationSystem/GBR/GBR_2011_EN.pdf) reveals that attainment of GCSE or A-levels corresponds to columns 5 and 6.

**Table 4:** Means and 95% confidence intervals, knowledge scores by condition.

	Icon array with arrows (*n*=313)	Icon array with gradient (*n*=342)	Icon array with shading (*n*=331)	Any icon array (*n*=986)	No icon array (*n*=314)
**Gist knowledge**					
Breast cancer more likely than ovarian cancer (max score: 4)	2.83 (2.68 – 2.97)	2.87 (2.74 – 3.01)	2.97 (2.83 – 3.11)	2.89 (2.81 – 2.97)	2.71 (2.56 – 2.86)
Breast cancer risk is increased (max score: 4)	3.20 (3.07 – 3.33)	3.18 (3.05 – 3.30)	3.24 (3.11 – 3.36)	3.21 (3.13 – 3.28)	3.10 (2.97 – 3.23)
Ovarian cancer risk is increased (max score: 4)	3.09 (2.98 – 3.21)	2.92 (2.80 – 3.05)	2.89 (2.76 – 3.02)	2.96 (2.89 – 3.04)	2.92 (2.79 – 3.05)
**Verbatim knowledge**					
Verbatim knowledge measure (max score: 8)	3.77 (3.55 – 3.99)	3.67 (3.47 – 3.88)	3.82 (3.61 – 4.02)	3.75 (3.63 – 3.87)	3.15 (2.96 – 3.34)
Frequency questions only (max score: 4)	1.84 (1.72 – 1.96)	1.92 (1.80 – 2.03)	1.96 (1.85 – 2.08)	1.91 (1.84 – 1.98)	1.58 (1.47 – 1.69)
Percentage questions only (max score: 4)	1.93 (1.81 – 2.06)	1.75 (1.64 – 1.87)	1.85 (1.74 – 1.97)	1.84 (1.78 – 1.91)	1.57 (1.46 – 1.68)

**Table 5:** ANOVAs on knowledge scores. Non-parametric ANOVA on ranks (Kruskal-Wallis tests) were used for gist knowledge scores due to non-normal residuals. * = *p* < 0.05; ** = *p* < 0.01; *** = *p* < 0.001.

	Test statistic (*X*^*2*^ (gist) *or* *F* (verbatim))	*df*	*p*	*η*^2^
**Gist knowledge**				
Breast cancer more likely than ovarian cancer	7.78	3	0.051	0.004
Breast cancer risk is increased	5.09	3	0.166	0.002
Ovarian cancer risk is increased	4.63	3	0.20	0.001
**Gist knowledge: any icon array vs. no icon array**				
Breast cancer more likely than ovarian cancer	5.03	1	0.025 *	0.003
Breast cancer risk is increased	4.55	1	0.033 *	0.003
Ovarian cancer risk is increased	0.36	1	0.55	−0.0005
**Verbatim knowledge**				
Verbatim knowledge measure	8.51	3, 1296	<0.001***	0.019
Frequency questions only	8.26	3, 1296	<0.001***	0.019
Percentage questions only	6.98	3, 1296	<0.001***	0.016

Given the nonsignificant effects of format, we ran ‘two one-sided t-tests’ (TOST) equivalence tests (on ranks)^2^ rather than Dunnett’s post-hocs, testing for the *absence of an effect* exceeding *d* = .23 (*η*^2^ = 0.013). Knowledge that *BRCA1* pathogenic variant carriers have an increased risk of ovarian cancer was equivalent to the control for the gradient (*p* = .002) and shaded (*p* = .004) formats, and knowledge that breast cancer is more likely than ovarian cancer was equivalent to the control for the arrow format (*p* = .043). No other equivalence tests were significant.

**Fig. 3:**
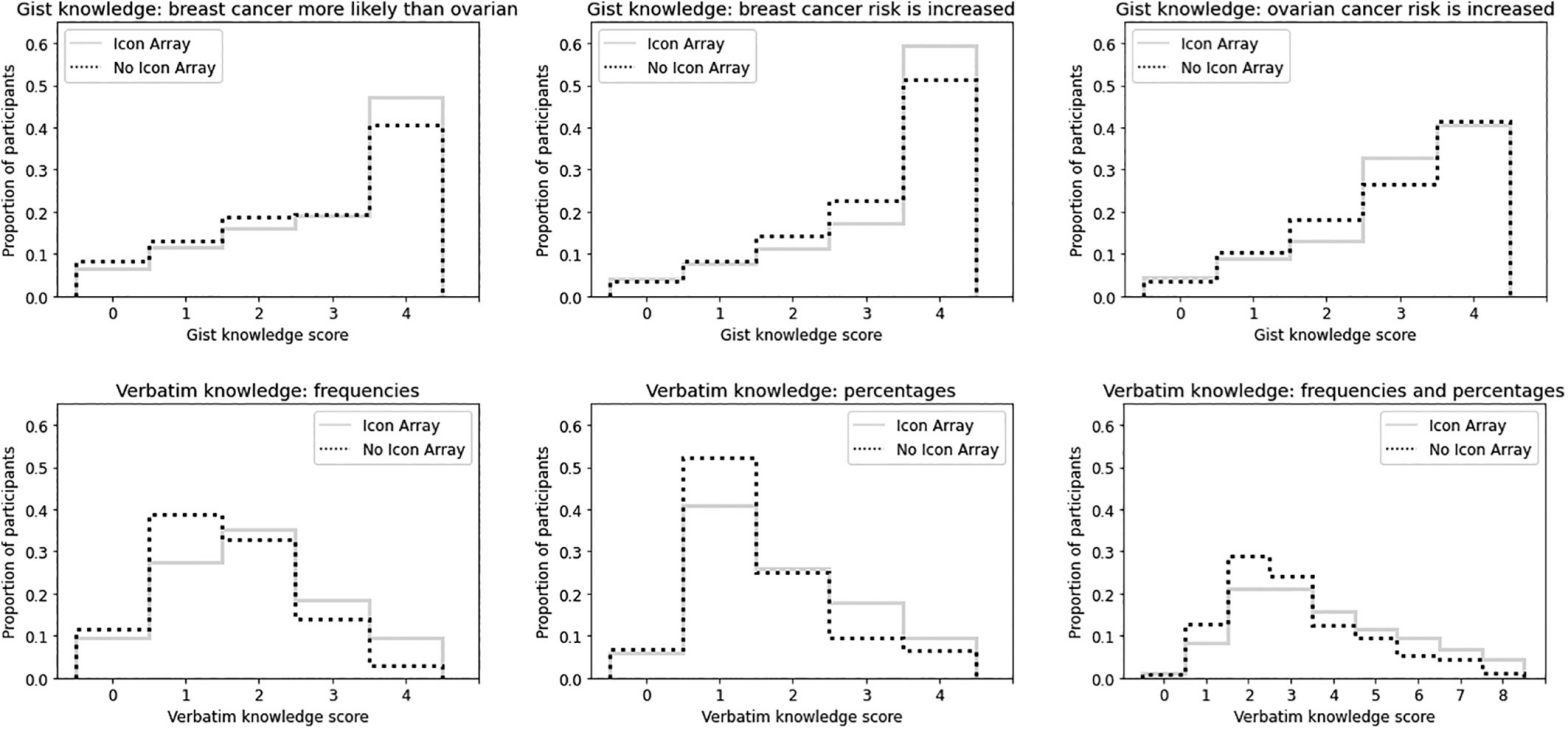
Histograms of gist and verbatim knowledge scores of participants for whom icon arrays were present vs. absent.

In the analyses of whether the presence vs. absence of an icon array facilitated gist knowledge, participants viewing icon arrays were slightly more likely than those viewing text alone to understand that, if unmanaged, Carla’s breast cancer risk would be higher than for the general population, and that she would be more likely to experience breast cancer than ovarian cancer (Table 4, Table 5; see also Fig. 3).

### 3.1 Exploratory analyses

#### 3.1.1 Impact of individual factors on gist knowledge

The finding that the presence of an icon array facilitated gist knowledge (for measures 1 and 2) was followed up with exploratory regressions to identify whether this was true for individuals of differing genders, numeracy levels, health literacy levels, and answers to the question about personal experience with cancer. These found that numeracy, subjective health literacy, objective health literacy, and female gender each predicted greater gist knowledge, but there were no interactions (Table S1). All binary and continuous covariates and the presence of an icon array predicted gist knowledge when included in the same model (Table 6; see Table S2 for correlation matrix).

**Table 6:** Exploratory regressions including *presence of icon array* and all binary and continuous covariates as independent variables. Tobit regression was used to address ceiling effects in the dependent variables.

*Independent variable*	β *Dependent variable:* *Gist measure 1* *(breast cancer more likely than ovarian cancer)* *R*^*2*^*= 0.162*	β *Dependent variable:* *Gist measure 2* *(breast cancer risk is increased)* *R*^*2*^*= 0.268*
Presence of icon array	0.358 (*z =* 2.311, *p* = 0.021*)	0.305 (*z =* 1.979, *p* = 0.048*)
Numeracy	0.438 (*z =* 5.688, *p* < 0.001***)	0.410 (*z =* 5.119, *p* < 0.001***)
Objective health literacy	0.339 (*z =* 8.845, *p* < 0.001***)	0.498 (*z =* 13.090, *p* < 0.001***)
Subjective health literacy	0.375 (*z =* 3.821, *p* < 0.001***)	0.506 (*z =* 5.254, *p* < 0.001***)
Gender (*Female*)	0.290 (*z =* 2.113, *p* = 0.035*)	0.404 (*z =* 2.935, *p* = 0.003**)
Personal experience with cancer	0.336 (*z =* 2.224, *p* = 0.026*)	0.299 (*z =* 1.970, *p* = 0.049*)

Aligned ranks ANOVAs investigating interactions between education (below upper secondary, upper secondary, or above upper secondary) and the presence vs. absence of an icon array for the three gist knowledge measures found that higher education levels predicted greater gist knowledge (Table 7; means and confidence intervals reported in Table S3). These also revealed interactions for two gist knowledge measures, with the lowest-education participants deriving more benefit from the presence of an icon array than participants who had completed secondary education (*η*^2^_*p*_ = .013 and .008, respectively; Fig. 5).

**Table 7:** Aligned ranks ANOVAs investigating interactions between education (below upper secondary, upper secondary, or above upper secondary) and the presence vs. absence of an icon array for the three gist knowledge measures.

	*F*	*df*	*p*	*η*_*p*_^2^
***Main effect of education***				
DV: Gist knowledge measure 1 (breast cancer more likely than ovarian cancer)	37.93	2, 1294	<.001***	.055
DV: Gist knowledge measure 2 (breast cancer risk is increased)	56.12	2, 1294	<.001***	.080
DV: Gist knowledge measure 3 (ovarian cancer risk is increased)	11.27	2, 1294	<.001***	.017
***Main effect of presence (vs. absence) of icon array***				
DV: Gist knowledge measure 1 (breast cancer more likely than ovarian cancer)	4.96	1, 1294	.026*	.004
DV: Gist knowledge measure 2 (breast cancer risk is increased)	32.14	1, 1294	<.001***	.024
DV: Gist knowledge measure 3 (ovarian cancer risk is increased)	9.53	1, 1294	.002**	.007
***Interaction effects***				
DV: Gist knowledge measure 1 (breast cancer more likely than ovarian cancer)	2.13	2, 1294	.119 (n.s.)	.003
DV: Gist knowledge measure 2 (breast cancer risk is increased)	8.64	2, 1294	<.001***	.013
DV: Gist knowledge measure 3 (ovarian cancer risk is increased)	5.21	2, 1294	.006**	.008

* = p < 0.05; ** = p < 0.01; *** = p < 0.001.

**Fig. 4:**
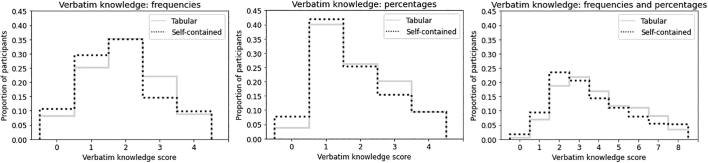
Histograms of verbatim knowledge scores of participants who viewed icon arrays with the self-contained vs. tabular layout.

**Fig. 5:**
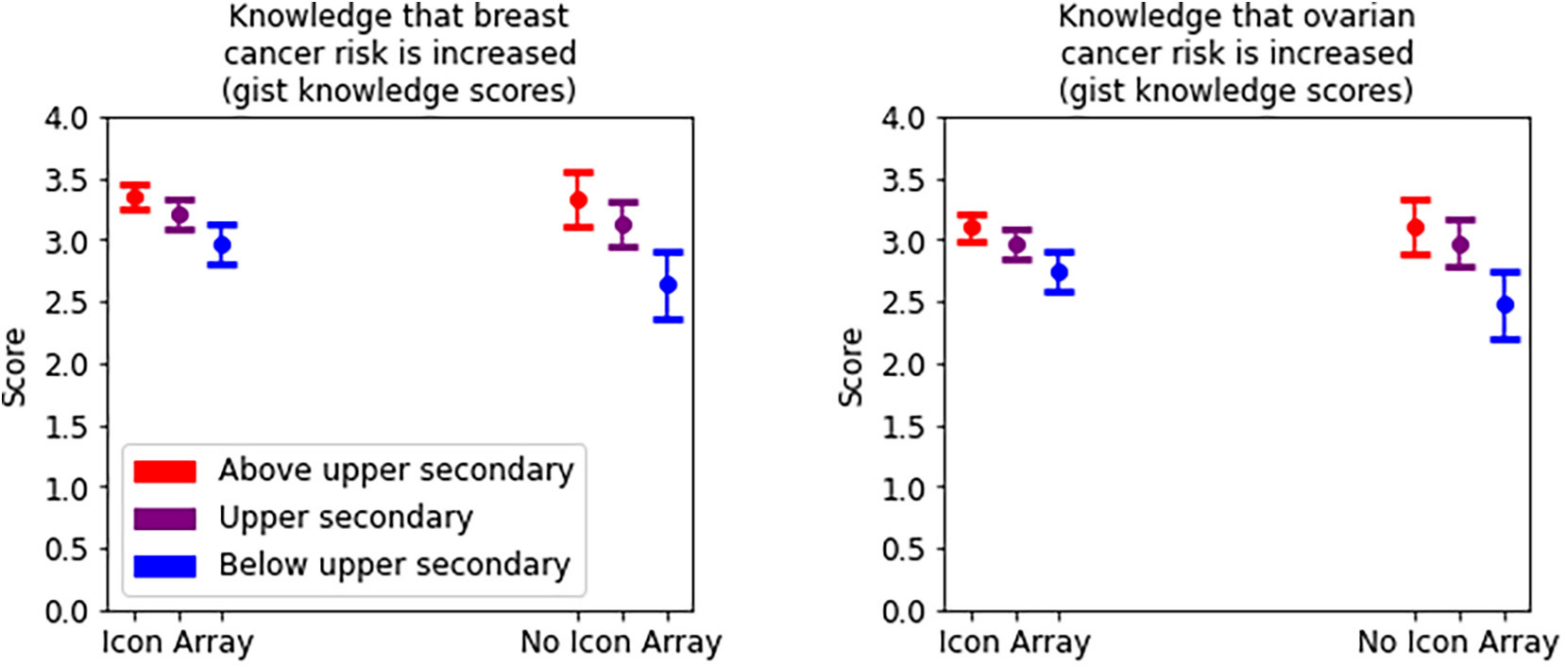
Interactions between icon array presence and highest level of education achieved, with 95% confidence intervals.

#### 3.1.2 Impact of icon arrays on verbatim knowledge

ANOVAs evaluating the effect of format on verbatim knowledge are reported in Table 4, Table 5; see also Fig. 3. Dunnett’s post-hocs showed that verbatim knowledge was higher than in the control condition for each condition (*p* < 0.01). Verbatim knowledge was slightly higher among icon arrays in the tabular (*M* = 3.89, CI = 3.73 – 4.06) vs. self-contained (*M* = 3.61, CI = 3.43 – 3.78) layout, *t*(981.3) = 2.33, *p* = .020, *d* = 0.15 (see Fig. 4.)

Item-wise analysis suggested that ovarian risk estimates (frequencies) were slightly higher among icon arrays in the tabular layout (*M* = 3.47 out of 8 women, CI = 3.32 – 3.62) than in the self-contained layout (*M* = 3.24, CI = 3.09 – 3.39), *t*(931.7) = 2.1, *p* = .033, *d* = 0.14. The same was true of carrier breast risk estimates (percentages), tabular: *M* = 63.3% (CI = 61.5% – 65.0%), self-contained: *M* = 60.3% (CI = 58.3% – 62.3%), *t*(965.46) = 2.2, *p* = 0.027, *d* = 0.14.

#### 3.1.3 Impact of icon arrays on subjective recall, subjective understanding, and subjective risk assessment and attitudes towards risk information

Ratings of how easy it was to recall the information (‘subjective recall’) exhibited a main effect of format, F(3, 1296) = 3.65, *p* = .012, *η*^2^ = 0.008, with Dunnett’s post-hocs suggesting it felt easier in the arrow (*M =* 3.86, CI = 3.70 – 4.02, *p* = 0.012) and shaded (*M* = 3.85, CI = 3.69 – 4.02, *p* = 0.013) conditions than in the control condition (*M* = 3.53, CI = 3.37 – 3.68). Ratings of how easy it was to understand the information (‘subjective understanding’) exhibited a ceiling effect; neither ANOVA nor a Kruskal-Wallis test found differences between groups (*p* = .77 and .95, respectively). There also was no effect of format on attitudes towards risk information. T-tests did not find differences between tabular and self-contained layouts for subjective recall, subjective understanding, or attitudes towards risk information.

For breast cancer risk, a Kruskal–Wallis test found a small main effect of format on participants’ subjective risk assessments, *X*^*2*^ = 9.83, df=3, *p* = .020, *η*^2^ = 0.005. Tukey’s post-hocs on mean ranks suggested risk was perceived as higher in the arrow (*M* = 5.70, CI = 5.57 – 5.83) condition than in the control condition (*M* = 5.51, CI = 5.39 – 5.63), *p*_*adj*_ = .019. There was no effect of format on assessments of ovarian cancer risk (across all conditions: *M* = 5.04, CI = 4.98 – 5.10).

#### 3.1.4 Analysis of whether risks to carriers were overestimated or underestimated

Verbatim knowledge questions that asked participants to state a carrier’s risks as a frequency were scored as correct if they were within the range shown on the report, as underestimates if they were below it, and as overestimates if they were above it^3^. 97.8% (437/447) of incorrect answers about a carrier’s breast cancer risk were underestimates, as were 60.9% (199/327) of incorrect answers about a carrier’s ovarian cancer risk. When asked to state the carrier’s risk as a percentage, 91.2% (562/616) of incorrect answers about a carrier’s breast cancer risk were underestimates, compared to 42.5% (234/550) of incorrect answers about a carrier’s ovarian cancer risk.

## 4 Discussion and conclusion

### 4.1 Discussion

Previous research had established that icon arrays often facilitate risk comprehension, but there has been little investigation of the effect of using them to communicate a risk range (e.g. ‘65%-79%’). We aimed to determine whether particular approaches to doing so had advantages over others when attempting to increase basic ‘gist knowledge’ about *BRCA1* risk. We also wanted to explore whether these approaches affected understanding of the specific amount of risk that carriers face (‘verbatim knowledge’), and whether they might help some subgroups of individuals more than others.

Our analyses did not find a clear benefit (or detriment) of any particular approach to communicating a range. However, it was encouraging that participants shown icon arrays with ranges scored better on two of three gist knowledge measures than those shown text alone, and also exhibited better verbatim knowledge. Although the increase in graphical complexity required to communicate a range could theoretically have increased ‘cognitive load’[58] to the point that including visualizations became counterproductive, we did not find this to be the case. However, these analyses were exploratory and their effect sizes were small.

Presenting icon arrays in the ‘tabular’ layout improved overall verbatim knowledge as well. An item-wise analysis suggested that for some questions on which our study population tended to underestimate the risk, participants viewing the ‘tabular’ layout answered more accurately. This is in line with research finding that presenting information in tabular ‘fact boxes’ facilitates comprehension[[59], [60], [61], [62]].

Ideally, patient-centered reports could serve as a tool that some patients may use to help communicate their results to family members or relatives. Correct interpretation of genetic risk by all family members facilitates communication of results within the family, which is important for cascade testing purposes. For these reasons we felt it was important not to restrict our study only to individuals assigned female at birth. However, this means it is unclear to what degree our findings generalize to real-world test recipients. Our study population tended to underestimate breast cancer risks, whereas these risks are often overestimated by women with family histories of breast cancer[44,63]. Encouragingly, icon arrays have been found to result in more accurate interpretations among women with familial breast cancer risks, including women who overestimate their risk[43,44]; this provides some hope that our findings for ‘ranged’ icon arrays may generalize as well. Due to the difference between our study population and women with known familial cancer risks, however, our findings likely generalize best to individuals who do not have a known family history of cancer, or who otherwise underestimate their risk. Exploratory analyses also suggested that, for gist knowledge measures 2 and 3, the presence of icon arrays was most beneficial for participants who had not completed secondary education. This suggests that, even when communicating the basic facts that breast and ovarian cancer risk is increased, routine use of icon arrays may be particularly helpful for this cohort. Our exploratory regressions also found that numeracy and health literacy were stronger predictors of gist knowledge than the presence of an icon array, underscoring the importance of following best practices for genetic risk communication that go beyond visualizations alone (see Conclusion).

Compared to the control group, participants viewing the arrow and shaded formats also reported that risk information was easier to recall. The arrow format also avoids a misconception arising in our qualitative work: one participant viewing the shaded format misinterpreted the shaded women as women who had not undergone treatment. That said, participants in the arrow condition were the only group whose subjective risk assessment was higher than for participants in the control condition, perhaps because in the visualization, individuals within the range appeared in solid black rather than in a more ‘uncertain’ gray or gradient. Healthcare providers who do not wish to use visualizations that influence patients’ subjective risk assessments may wish to bear this in mind.

#### 4.1.1 Limitations

This research did not evaluate perceptions of the level of uncertainty per se, nor include a comparison group who was provided a point estimate without a range. Alternative approaches worth testing in more detail include the ‘step-by-step’ coloring[19] of Raphael et al., or replacing our arrows with a diamond, fan, violin or density band[64,65], which might more effectively communicate that the true value is likelier to be toward the center than the edges of the range.

We also did not collect data on biological sex, graphical literacy, or experience with breast/ovarian cancer specifically [30,66]. Although the decision not to include percentages on the reports was deliberate (see Methods), this limits the conclusions that can be drawn from the ‘verbatim’ questions that requested responses as percentages; the “out of 8” framing also makes interpretation more difficult. It may be valuable for future work to test whether these findings generalize to current patients and participants with low graphical literacy, and whether low graphical literacy can be addressed with interventions previously shown to help this cohort, such as the inclusion of more detailed explanations[66].

### 4.2 Innovation

To our knowledge, this is the first study empirically comparing the effects of different ways of visualizing imprecision within an icon array, and the first study to do so in a *BRCA* risk context. This is striking given how commonly icon arrays and ranges are used to communicate medical risks, and we hope to see more future work in this area. It also represents a case in which patient involvement led to the study of visualizations which would not have been considered otherwise, pointing to the benefits of patient involvement in stimuli development.

### 4.3 Conclusion

Ultimately, our findings did not reveal a clear winner among the specific approaches we tested for illustrating a range in an icon array. However, they do suggest that presenting an icon array with a range may communicate risk more effectively than no icon array at all. We suggest that healthcare providers who prefer to present risks only as ranges should not shy away from using icon arrays, and we provide three reasonable options for doing so. Visualizations should be supplemented with genetic risk communication best practices such as keeping language to an 8th grade level or below, avoiding relative risk statements such as "your risk is tripled", presenting risks in the same numerical format to eliminate the need for mental mathematics, using plain language addressed towards the patient, and providing trusted resources where patients can turn for more information and support (see [[67], [68], [69]] for further recommendations). Our findings also point toward a possible benefit of displaying multiple icon arrays in a tabular layout, and provide a starting place for further investigations.

## Data accessibility

Code and data for all analyses is available at https://osf.io/y5fm6.

I confirm all patient/personal identifiers have been removed or disguised so the patient/person(s) described are not identifiable and cannot be identified through the details of the story.

## Declaration of Competing Interest

The Authors declare that there is no conflict of interest.

## Appendix A Supplementary data

Supplementary material 1 Image 4

Supplementary material 2 Image 5

Supplementary material 3 Image 6

## Footnotes

1 Cronbach’s alpha is a special case of ω_total_: when tau equivalence is met, ω_total_ will yield the same result as Cronbach’s alpha, but ω_total_ has the flexibility to handle situations in which items have skewed distributions. While there are no universal criteria for the interpretation of ω_total_, values of less than .5 for Cronbach’s alpha are below most commonly cited criteria for scale acceptability. That said, there is no consensus on exactly what suitable thresholds for Cronbach’s alpha should be or indeed whether thresholds should be used at all, and values this low can be expected for reasonable scales in some contexts[71].

2 Although the preregistration stated that non-parametric alternatives may be employed if data violates ANOVA assumptions to such a degree that ANOVAs would be inappropriate, we neglected to state this for the post-hoc tests; nonparametric post-hocs (i.e., on ranks) were conducted as well.

3 If a participant provided a range rather than a single number, their answer was treated as the midpoint of the range. Ranges equal or close to the ranges shown on the report were therefore scored as correct.
