# Supplementary material for: Investigating the presentation of uncertainty in an icon array: A randomized trial
Source: PEC Innov. 2021 Oct 30;1:100003. doi: 10.1016/j.pecinn.2021.100003 (PMC9731905; doi:10.1016/j.pecinn.2021.100003)
Supplement: Supplementary file 1 — Supplementary material 1 [file mmc1.docx]

*Types of recall errors for participants who provided a range when asked how many women they expected to experience breast and ovarian cancer*

In the visualisations used in this study, endpoints were placed such that the proportion of individuals expected to experience the outcome lay to the left of the visual representation of the range, and the proportion expected not to experience the outcome lay to the right, with the remainder falling in between. This had the advantage of allowing the width of the range to be accurately illustrated, but may also have caused confusion. For example, in Figure 2a, some individuals may have expected the arrow to hover above the set of reasonable possibilities for the number of women who would experience breast cancer (“5 to 7”), rather than to point to the minimum and maximum number of women likely to experience the outcome. Answers to the questions that asked participants to recall how many women, out of 8, would be expected to experience breast and ovarian cancer may shed some light upon the most common interpretations. For breast cancer, of the 299 who both were shown an icon array and provided an unambiguous range rather than a single number, 258 (86%) correctly recalled 5-7, 14 (5%) accidentally recalled the range for ovarian cancer, and 7 (2%) recalled 6-7, suggesting possible confusion related to the placement of the endpoints, with the remainder making other errors. For ovarian cancer, of 185 who were shown an icon array and provided an unambiguous range, 105 (57%) correctly recalled 2-5, 14 (8%) accidentally recalled the range for breast cancer, and 11 (6%) recalled 3-5.

These results suggest that only a small minority of participants may have misinterpreted the range as a result of the placement of the endpoints. However, the fact that the stimuli stated the ranges explicitly makes it difficult to know how individuals would have interpreted the ranges depicted in the visualizations if there had not been accompanying labels or text; this is a potential avenue for future research.

*Subgroup analyses*

Among the subgroups of individuals who scored in the bottom half of the sample with respect to objective health literacy, subjective health literacy, and numeracy, there were no significant differences between participants in different format conditions, or those for whom icon arrays were present vs. absent. There were also no significant differences for those shown the tabular vs. nontabular layout.

*Exploratory Tukey’s post-hoc tests*

Besides the differences reported in sections 3.1.2 and 3.1.3 detected by Dunnett’s post-hocs, Tukey’s post-hocs revealed no additional differences between formats for verbatim knowledge, subjective recall, subjective understanding, subjective risk assessment, or attitudes towards risk information.
