# Supplementary material for: Investigating the presentation of uncertainty in an icon array: A randomized trial
Source: PEC Innov. 2021 Oct 30;1:100003. doi: 10.1016/j.pecinn.2021.100003 (PMC9731905; doi:10.1016/j.pecinn.2021.100003)
Supplement: Supplementary file 2 — Supplementary material 2 [file mmc2.docx]

**Figure S1.** The genetic report, shown here with the arrow (marked endpoints) uncertainty format and the tabular array layout. Readers developing similar communications may wish to consider clearer alternatives to the phrase “women with alterations like yours in *BRCA1* (with no treatment)”, such as “medically unmanaged women with alterations like yours in *BRCA1*.”

**
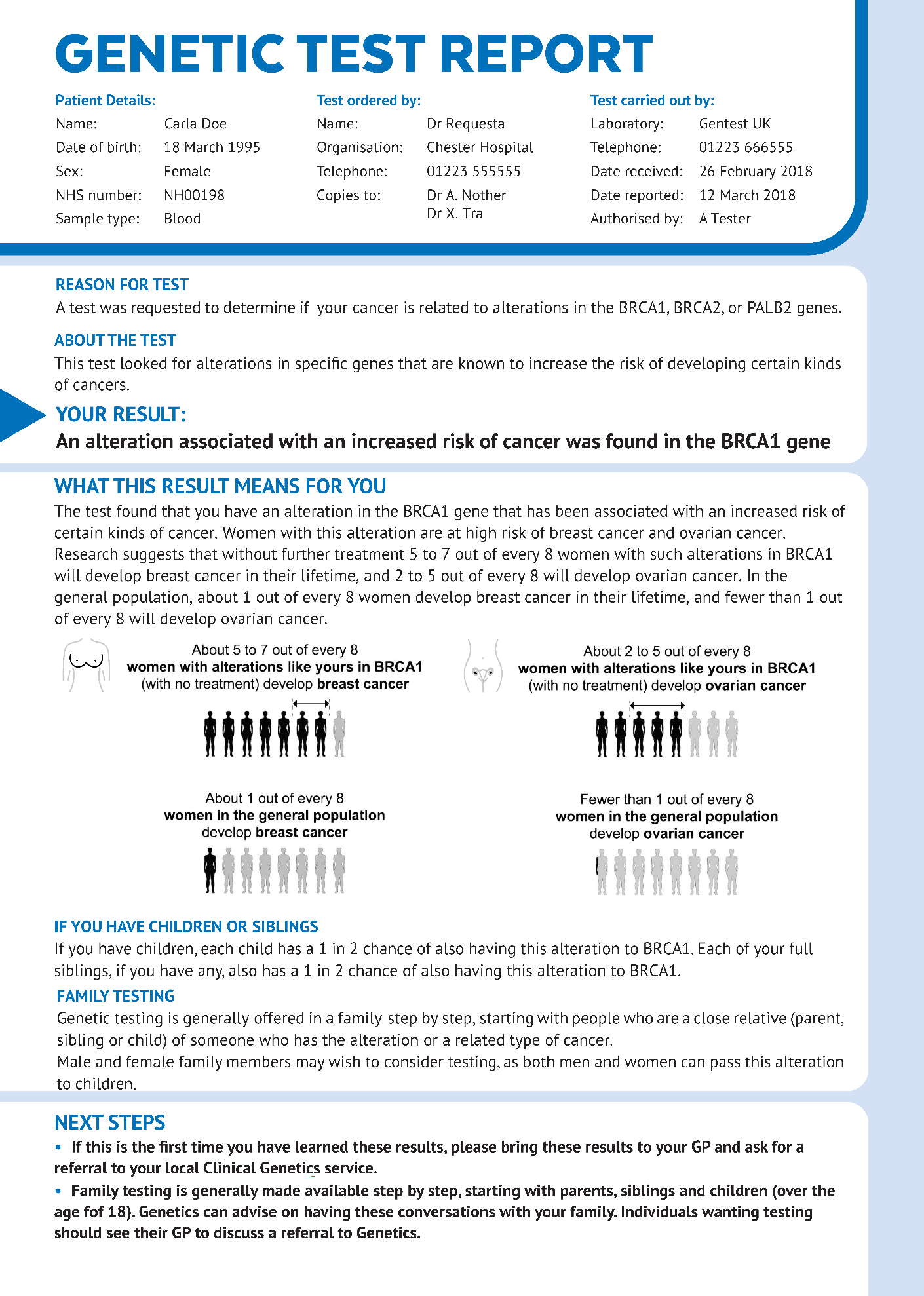
**
