# Supplementary material for: Investigating the presentation of uncertainty in an icon array: A randomized trial
Source: PEC Innov. 2021 Oct 30;1:100003. doi: 10.1016/j.pecinn.2021.100003 (PMC9731905; doi:10.1016/j.pecinn.2021.100003)
Supplement: Supplementary file 3 — Supplementary material 3 [file mmc3.docx]

**Table S1.** Exploratory regression models of the form Y = β_0_ + β_1_X_1_ + β_2_X_2_ + β_3_X_1_X_2_ + ε*,* where X_1_ is coded 0 when an icon array was present and 1 when an icon array was absent, conducted to test for interactions between *presence of icon array* and all binary and continuous covariates. Tobit regression was used to address ceiling effects in the dependent variables. * = p < 0.05; ** = p < 0.01; *** = p < 0.001.

| *Y* | *X_2_* | *R^2^* | β_1_ | β_2_ | β_3_ |
| --- | --- | --- | --- | --- | --- |
|  |  |  |  |  |  |
| Gist measure 1 (breast cancer more likely than ovarian cancer) | 0  (no X_2_ term) | 0.004 | 0.382  (*z =* 2.237,  *p* = 0.025*) | - | - |
| Gist measure 2 (breast cancer risk is increased) | 0  (no X_2_ term) | 0.002 | 0.357  (*z =* 1.929,  *p* = 0.054) | - | - |
| Gist measure 1 (breast cancer more likely than ovarian cancer) | Numeracy | 0.056 | 0.002  (*z =* 0.007,  *p* = 0.99) | 0.573  (*z =* 3.957,  *p* < 0.001***) | 0.224  (*z =* 1.316,  *p* = 0.19) |
| Gist measure 2 (breast cancer risk is increased) | Numeracy | 0.071 | -0.104  (*z =* -0.287,  *p* = 0.77) | 0.709  (*z =* 4.406,  *p* < 0.001***) | 0.271  (*z =* 1.420,  *p* = 0.16) |
| Gist measure 1 (breast cancer more likely than ovarian cancer) | Objective health literacy | 0.132 | -0.009  (*z =* -0.027,  *p* = 0.98) | 0.395  (*z =* 5.600,  *p* < 0.001***) | 0.103  (*z =* 1.279,  *p* = 0.20) |
| Gist measure 2 (breast cancer risk is increased) | Objective health literacy | 0.232 | -0.082  (*z =* -0.271,  *p* = 0.79) | 0.559  (*z =* 7.966,  *p* < 0.001***) | 0.119  (*z =* 1.470,  *p* = 0.14) |
| Gist measure 1 (breast cancer more likely than ovarian cancer) | Subjective health literacy | 0.056 | -0.412  (*z =* -0.441,  *p* = 0.66) | 0.648  (*z =* 3.112,  *p* = 0.002**) | 0.205  (*z =* 0.864,  *p* = 0.39) |
| Gist measure 2 (breast cancer risk is increased) | Subjective health literacy | 0.082 | 0.324 (*z =* 0.329,  *p* = 0.74) | 1.119  (*z =* 5.038,  *p* < 0.001***) | 0.06  (*z =* 0.024,  *p* = 0.98) |
| Gist measure 1 (breast cancer more likely than ovarian cancer) | Gender (*Female*) | 0.012 | 0.554  (*z =* 2.276,  *p* = 0.023*) | 0.664  (*z =* 2.247,  *p* = 0.0246*) | -0.326  (*z =* -0.957,  *p* = 0.34) |
| Gist measure 2 (breast cancer risk is increased) | Gender (*Female*) | 0.018 | 0.520  (*z =* 2.009,  *p* = 0.045*) | 0.887  (*z =* 2.789,  *p* = 0.005**) | -0.317  (*z =* -0.863,  *p* = 0.39) |
| Gist measure 1 (breast cancer more likely than ovarian cancer) | Personal experience with cancer | 0.012 | 0.282  (*z =* 1.407,  *p* = 0.16) | 0.159  (*z =* 0.487,  *p* = 0.63) | 0.369  (*z =* 0.977,  *p* = 0.33) |
| Gist measure 2 (breast cancer risk is increased) | Personal experience with cancer | 0.009 | 0.393  (*z =* 1.819,  *p* = 0.07) | 0.574  (*z =* 1.605,  *p* = 0.11) | -0.119  (*z =* -0.288,  *p* = 0.77) |

**Table S2.** Correlation matrix of all independent variables in Table 7, and correlations between dependent and independent variables.

| ***Independent variable*** | Presence of icon array | Numeracy | Objective health literacy | Subjective health literacy | Gender (*Female*) | Personal experience with cancer |
| --- | --- | --- | --- | --- | --- | --- |
| Numeracy | 0.01 | - | - | - | - | - |
| Obj. health literacy | 0.02 | 0.38 | - | - | - | - |
| Subj. health literacy | 0.00 | 0.16 | 0.31 | - | - | - |
| Gender (*Female*) | -0.02 | -0.11 | 0.09 | 0.11 | - | - |
| Experience w/cancer | -0.01 | -0.06 | 0.04 | 0.04 | 0.09 | - |
| ***Dependent variable*** |  |  |  |  |  |  |
| Gist measure 1 | 0.06 | 0.23 | 0.36 | 0.23 | 0.09 | 0.09 |
| Gist measure 2 | 0.04 | 0.26 | 0.48 | 0.28 | 0.13 | 0.09 |

**Table S3.** Means and 95% confidence intervals, gist knowledge scores by education level.

|  | Above upper secondary (*n*=465) | Upper secondary (*n*=287) | Below upper secondary  (*n*=548) |
| --- | --- | --- | --- |
|  |  |  |  |
| 1: Breast cancer more likely than ovarian cancer (max score: 4) | 3.05  (2.94 – 3.17) | 2.83 (2.72 – 2.94) | 2.53 (2.38 – 2.68) |
| 2: Breast cancer risk is increased  (max score: 4) | 3.35  (3.25 – 3.44) | 3.19 (3.09 – 3.29) | 2.89 (2.75 – 3.03) |
| 3: Ovarian cancer risk is increased (max score: 4) | 3.10 (3.00 – 3.20) | 2.97 (2.87 – 3.06) | 2.67 (2.55 – 2.83) |
